# Eating disorders and social media use among college students in Japan and China: a brief cross-sectional survey

**DOI:** 10.1186/s40337-024-00999-w

**Published:** 2024-04-04

**Authors:** Yijing Bai, Noriko Numata, Eiji Shimizu

**Affiliations:** 1 United Graduate School of Child Development, Osaka University, Kanazawa University, Hamamatsu University School of Medicine, Chiba University and University of Fukui, at the Chiba Campus, Chiba, 260-8670 Japan; 2 Research Center for Child Mental Development, Chiba University, 1-8-1 Inohana, Chuo-ku, Chiba, 260-8670 Japan; 3 Department of Cognitive Behavioral Physiology, Chiba University Graduate School of Medicine, 1-8-1 Inohana, Chuo-ku, Chiba, 260-8670 Japan

**Keywords:** Eating disorders, Adolescence, Social media, Body esteem, International comparison, Asian countries, Gender differences

## Abstract

**Background:**

In recent years, new forms of media-social networking services (SNS)-such as Facebook and Sina Weibo have spread rapidly. Greater SNS use has been associated with greater body dissatisfaction, which in turn, is related to greater eating disorder (ED) symptom severity. In this study, we (1) investigated the relationships between ED tendencies, SNS use intensity, and body esteem and (2) examined the mediating role of body esteem in the relationship between SNS use intensity and ED tendencies among Japanese and Chinese students.

**Methods:**

A total of 564 Japanese and Chinese college students aged 18–22 years were surveyed on their age and BMI, including self-filling questions from the Japanese and Chinese versions of the Eating Attitudes Test (EAT-26), SNS Intensity Scale, Body Esteem Scale for Adolescents and Adults (BESAA), Patient Health Questionnaire-9 (PHQ-9), and Generalized Anxiety Disorder-7 (GAD-7) scale.

**Results:**

The proportion of students with a score of ≥ 20 on the EAT-26 was 15.8% (Japanese, 14.7%; Chinese, 17.0%). The number of participants with scores ≥ 20 on the EAT-26 was significantly higher than ever before, both in Japan and China. Chinese students reported greater body esteem than Japanese students, as well as a stronger association of SNS use intensity with body esteem. Among Japanese students, EAT-26 scores were unrelated to SNS Intensity Scale scores but had a weak negative correlation with BESAA scores; therefore, body esteem did not mediate the relationship between SNS use intensity and ED tendencies. Among Chinese students, scores on the SNS Intensity Scale and BESAA had a weak correlation with EAT-26 scores, and SNS use intensity reduced ED tendencies through greater body esteem.

**Conclusions:**

It is important to consider the way users engage with SNS, in addition to the SNS use intensity. Improving body esteem may reduce the risk of ED. Furthermore, it is necessary to include men in the discussion on ED in the future.

## Abstract (plain language summary)

We investigated the relationships between eating disorder (ED) tendencies, social networking service (SNS) use intensity, and body esteem among Japanese and Chinese students, and compared the findings. We surveyed 564 Japanese and Chinese students regarding basic demographic information, ED tendencies, SNS use, body esteem, depression, and anxiety. The results showed that the number of participants with scores ≥ 20 on the Eating Attitudes Test was significantly higher than ever before, both in Japan and China. In Japanese students, body esteem affected ED tendencies, while SNS use intensity did not. In Chinese students, SNS use intensity reduced ED tendencies through greater body esteem. There were no gender differences in Eating Attitudes Test scores among Japanese students. This study provides evidence for the relationship between ED tendencies, SNS use, and body esteem in Asian countries.

## Background

Eating disorders (ED) are mental illnesses in which abnormalities in eating behavior are observed. The Diagnostic and Statistical Manual of Mental Disorders, Fifth Edition (DSM-5) recognizes anorexia nervosa (AN), bulimia nervosa (BN), and binge eating disorder (BED) as the most prominent ED.

In a review of ED from 2000 to 2018, the lifetime prevalence of AN was 1.4% (0.1–3.6%) in women and 0.2% (0–0.3%) in men; that of BN was 1.9% (0.3–4.6%) in women and 0.6% (0.1–1.3%) in men; and that of BED was 2.8% (0.6–5.8%) in women and 1.0% (0.3–2.0%) in men [1]. Specific to Japan, the prevalence of AN was 0.2% and that of BN was 2.9% in 2010 [2]. As for ED among college students in China, the prevalence of AN was 1.1%, BN was 3.0%, and BED was 3.5% in 2014 [3].

Important sociocultural factors responsible for the development of ED are consumerism, which accompanies high economic growth, and the influence of Western culture, wherein thinness is popularized through the media. In recent years, new forms of media-social networking services (SNS)-such as Facebook and Instagram have spread rapidly, making it easy to share personal information, including photos. According to the official 2018 report from the China Internet Network Information Center, WeChat (utilization rate:85.5%) and Sina Weibo (utilization rate: 37.1%) were the most popular social medias [4]. Sina Weibo combines features and functions of Twitter, Facebook, and bulletin board systems [5, 6].

In an experimental study, female college students from the U.S. who spent time on Facebook reported greater concerns about their shape and weight and a higher state of anxiety compared to a control group who viewed a neutral website [7]. Body dissatisfaction occurs when there is a discrepancy between a person’s assessment of their actual body and an ideal body. This is generally attributed to social factors, with social media being the most common cause [8]. Studies on high school girls have found that internet usage is related to appearance comparisons and body dissatisfaction [9].

In studies on body dissatisfaction and ED among Chinese students in Hong Kong, those with increased exposure to Western cultural influences had increased body dissatisfaction and ED [10, 11]. Moreover, exposure to Western ideas and social change has been associated with increased body dissatisfaction among Chinese women, similar to the findings among women in Hong Kong [12, 13]. A survey of female university students in China found that 77.1% were dissatisfied with their bodies, and 75.6% had strict dietary restrictions [14]. Negative body image is a potential risk factor for ED.

Therefore, we hypothesized that students who reported greater SNS intensity are more likely to report lower body esteem and greater ED tendencies, and that body esteem would mediates the relationship between SNS intensity and ED tendencies.

Research on this topic has generally been limited to women. Both women and men are thought to be at a risk of ED tendencies, but men are more likely to hide their symptoms. Considering this, we assumed that the incidence of ED may be underestimated in men. Therefore, in this study, we included both men and women.

Kaye et al. found that the proportion of people with anxiety disorders is higher among those with ED than among the general population. In addition, anxiety disorders have been reported to precede ED [15]. Moreover, Zaider et al. showed that people with depression are more likely to develop diseases such as AN and BN [16]. Therefore, we used the Patient Health Questionnaire-9 (PHQ-9) and Generalized Anxiety Disorder-7 (GAD-7) scale to detect depression and anxiety as references.

Furthermore, research on the effects of SNS and ED has been mostly been conducted on Western women. In Asia, this research topic is still in its early stages, with relatively few empirical studies. Western media are influenced by the cultural context of emphasis on thin female bodies and weight control. China has a unique SNS platform and relative lack of access to Western media and influences compared to Japan. Under these circumstances, we chose to explore the relationship between SNS use and ED in Japan and China, as well as the differences between them. We hypothesized that Japanese students would report more ED tendencies and a greater association between SNS use intensity and ED tendencies than Chinese students.

Accordingly, we aimed to examine demographic data, such as gender and age, and the relationships between ED tendencies, SNS use, and body esteem among Japanese and Chinese students. The results of the surveys in Japan and China were compared to consider the differences between the two countries.

The results obtained in this study may help clarify the risk of ED in Japan and China and contribute to the development of preventive approaches by enabling the early identification of those prone to developing ED. Furthermore, we believe this study can deepen our understanding of the relationship between ED and SNS use.

## Method

### Design

A cross-sectional design was employed.

### Participants

We recruited 600 healthy participants nationwide from Japan and China via Cross Marketing Inc. (Tokyo, Japan), which engages in the online distribution and collection of survey content. The inclusion criteria were: (a) men and women aged 18–25, (b) students affiliated with any educational institution at the time of answering the questionnaire, and (c) not attending psychiatric clinic at the time when answering the questionnaire (regardless of illness). We provided an informed consent statement at the onset of the online survey and assumed that those who clicked “Next” had read it and agreed.

**Fig. 1:**
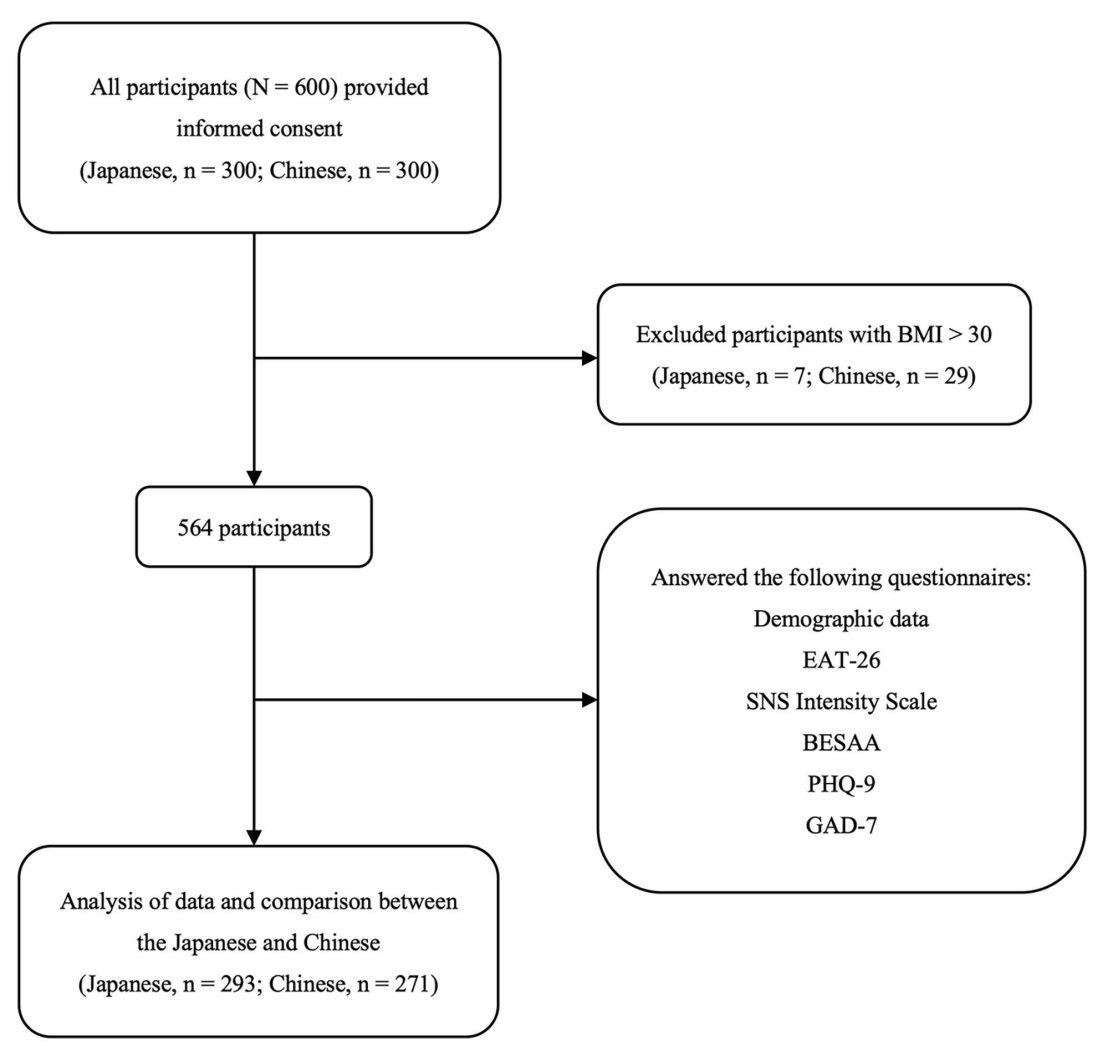
Flowchart of participant recruitment and selection

Thirty-six students (seven from Japan and twenty-nine from China) with a body mass index (BMI) > 30 kg/m^2^ were excluded as they came under the obese category as defined by the World Health Organization [17]. Finally, there were 564 valid responses (94% of the total; 293 from Japan, and 271 from China). Figure 1 provides details about the participant selection process.

### Measures

#### Eating attitudes Test-26 [18]

The Eating Attitudes Test-26 (EAT-26) was used to identify participants at high risk of ED. This 26-item instrument is scored on a 6-point Likert scale ranging from 1 (always) to 6 (never). The total score ranges from 0 to 78 points. An overall score of ≥ 20 indicates a risk of developing ED. In Japanese and Chinese, the Cronbach’s α was 0.939 and 0.913, respectively.

#### SNS intensity scale [19]

We used the Facebook Intensity Scale to measure the frequency of SNS use and attitudes toward SNS. The SNS that people frequently use differs depending on the country. In Japan, the most used SNS are Facebook, Instagram, and Twitter (changed to “X” in 2023); and in China, these are Weibo and WeChat [20]. 

This scale consists of two self-reported assessments of SNS behavior and six questions evaluating the attituded toward SNS by the raw scores on 5-point Likert scale. The total score of the SNS Intensity Scale is calculated by summing the raw scores on the 5-point Likert-type scale and the log (amount of time on SNS) and log (total SNS friends). Higher scores reflected greater SNS use intensity.

One behavioral item asks participants about the amount of time they spent on SNS: “In the past week, on average, approximately how many minutes per day have you spent on SNS?” The second behavioral item captures the number of friends on SNS: “About how many total SNS friends do you have?” Regarding attitudes toward SNS, we used six items reflecting emotional investment in SNS (e.g., “I feel I am part of the SNS community”). In Japanese and Chinese, the Cronbach’s α was 0.835 and 0.878, respectively.

#### Body esteem scale for adolescents and adults [21]

The Body Esteem Scale for Adolescents and Adults (BESAA) consists of 23 items across three factors: BES-appearance (e.g., “I like what I look like in pictures”), BES-weight (e.g., “Other people consider me good looking”), and BES-attribution (e.g., “I’m satisfied with my weight”). This instrument is scored on a 5-point Likert scale ranging from 0 (never) to 4 (always). Higher scores indicated higher body esteem. In Japanese and Chinese, the Cronbach’s α was 0.885 and 0.8, respectively.

#### The Patient Health Questionnaire-9 [22]

The Patient Health Questionnaire-9 (PHQ-9) assesses the frequency and severity of symptoms of depression using nine items on a 4-point Likert scale ranging from 0 (not at all) to 3 (nearly every day). The total score ranges from 0 to 27 points. In Japanese and Chinese, the Cronbach’s α was 0.927 and 0.88, respectively.

#### The generalized anxiety Disorder-7 [23]

The Generalized Anxiety Disorder-7 (GAD-7) measures the frequency and severity of generalized anxiety disorder symptoms using seven items on a 4-point Likert-scale ranging from 0 (not at all) to 3 (nearly every day). The total score rangs from 0 to 21 points. In Japanese and Chinese, the Cronbach’s α was 0.927 and 0.892, respectively.

### Procedure and statistical analysis

All statistical analyses were performed using SPSS Statistics 26.0 (IBM Corp., Armonk, NY, USA), and statistical significance was set at *p <* 0.05. A one-way analysis of variance was conducted to examine gender or country-based differences across variables. The Bonferroni test or Tamhane’s T2 test was used for all post hoc pair-wise comparisons between groups. Pearson’s correlation analyses were conducted to examine relationships between variables. Mediation analyses were conducted to test the mediating role of body dissatisfaction in the relationship between SNS use intensity and ED tendencies.

We used the PROCESS macro for SPSS to examine the direct effect of SNS use intensity on ED tendencies and the BESAA scores to determine the indirect mediating effect of body esteem in the association between SNS use intensity and ED tendencies. BMI and gender were included as covariates.

## Results

### Demographic characteristics and comparisons between countries and gender

Descriptive characteristics and results of the analysis of variance examining country and gender differences are presented in Table 1; post hoc analyses were performed for significant differences.

Among the Japanese students (*n =* 293), 48.8% were men (*n =* 143) and 51.2% were women (*n =* 150). The average age and BMI were 20.49 years and 21.22 kg/m^2^ for men, and 20.43 years and 20 kg/m^2^ for women, respectively.

Among the Chinese students (*n* = 271), 49.4% were men (*n =* 134) and 50.6% were women (*n =* 137). The average age and BMI were 20.63 years and 21.81 kg/m^2^ for men, and 20.36 years and 20.31 kg/m^2^ for women, respectively.

The proportions of participants’ EAT-26 total scores ≥ 20 by gender and country were as follows: Japanese men, 12.6% (18/143); Japanese women, 16.6% (25/150); Chinese men, 9.7% (13/134); and Chinese women, 24.1% (33/137). Chinese women (*M ± SD =* 12.91 ± 12.76) and Japanese women (*M ± SD =* 11.33 ± 12.73) had significantly higher scores on the EAT-26, and no gender differences were found in Japanese students.

Regarding SNS use intensity, Japanese men reported a range of 51 to 150 SNS friends and 31 min to two hours of SNS use per day, while Japanese women reported a range of 100 to 200 SNS friends and 31 min to two hours of SNS use per day. Chinese men reported a range of 101 to 200 SNS friends and 31 min to two hours of SNS use per day, while Chinese women reported a range of 100 to 200 SNS friends and one hours to three hours of SNS use per day. Chinese men (*M ± SD =* 24.51 ± 4.73) and women (*M ± SD =* 25.04 ± 5) reported higher scores on the SNS Intensity Scale than their Japanese counterparts.

BESAA scores were significantly higher among Chinese men (*M ± SD =* 52.13 ± 10.32) and women (*M ± SD =* 50.07 ± 12.45) than among Japanese men (*M ± SD =* 39.55 ± 12.04) and women (*M ± SD =* 31.83 ± 14.27).

PHQ-9 scores did not demonstrate group differences (*F* = 1.474; *p* = 0.221). However, on the GAD-7, Chinese women (*M ± SD =* 5.23 ± 4.63) scored higher than Japanese men.

**Table 1:** Descriptive characteristics and analysis of variance comparing the four groups

	Japanese men (*n* = 143)	Japanese women (*n* = 150)	Chinese men (*n* = 134)	Chinese women (*n* = 137)	F	*P*
Age	20.49 ± 1.17	20.43 ± 1.2	20.63 ± 1.13	20.36 ± 1.29	1.258	0.288
BMI (kg/m2)	21.22 ± 2.64^a^	20 ± 2.36^b^	21.81 ± 2.37^a^	20.31 ± 2.37^b^	16.271	< 0.001
EAT-26 score ≥ 20 (n)	18 (12.6%)	25 (16.6%)	13 (9.7%)	33 (24.1%)	/	/
EAT-26	8.41 ± 13.37^b^	11.33 ± 12.73^ab^	8.43 ± 9.49^b^	12.91 ± 12.76^a^	4.629	0.003
Total of SNS Intensity Scale	18.45 ± 5.58^b^	19.1 ± 5.83^b^	24.51 ± 4.73^a^	25.04 ± 5^a^	60.095	< 0.001
Number of SNS friends	3.66 ± 2.92^b^	4.30 ± 2.98^ab^	4.70 ± 2.06^a^	4.82 ± 2.05^a^	5.801	0.001
Time on SNS	3.11 ± 1.64^c^	3.87 ± 1.70^ab^	3.79 ± 1.08^b^	4.18 ± 1.16^a^	13.769	< 0.001
SNS attitudes	17.47 ± 5.25^b^	17.89 ± 5.54^b^	23.13 ± 4.69^a^	23.6 ± 4.93^a^	58.037	< 0.001
Total of BESAA	39.55 ± 12.04^b^	31.83 ± 14.27^c^	52.13 ± 10.32^a^	50.07 ± 12.45^a^	83.241	< 0.001
BES-appearance	18.57 ± 6.31^b^	14.87 ± 7.2^c^	22.18 ± 5.21^a^	22.6 ± 6.14^a^	47.316	< 0.001
BES-attribution	5.24 ± 4.54^b^	4.6 ± 4^b^	11.58 ± 4.26^a^	11.28 ± 4.15^a^	111.478	< 0.001
BES-weight	15.74 ± 5.28^b^	12.35 ± 5.93^c^	18.37 ± 4.88^a^	16.19 ± 5.71^b^	29.534	< 0.001
PHQ-9	5.6 ± 6.13	7.01 ± 6.58	6.56 ± 5.32	6.49 ± 5.09	1.474	0.221
GAD-7	3.48 ± 4.54^b^	4.76 ± 5.1^ab^	4.54 ± 4.26^ab^	5.23 ± 4.63^a^	3.552	0.014

Note: Values with different superscript letters a, b, c, and d are significantly different (*p* < 0.05). BMI: body mass index; EAT-26: Eating Attitudes Scale-26, SNS: social networking services; BESAA: Body-Esteem Scale for Adolescents and Adults; GAD-7: Generalized Anxiety Disorder scale-7, PHQ-9: Patient Health Questionnaire-9

### Correlations between the Japanese and Chinese students

We conducted a correlation analysis among the study variables, as shown in Tables 2 and 3. Specifically, we performed a correlation analysis between EAT-26, SNS Intensity Scale, and BESAA scores. Among Japanese students, BMI was negatively correlated with the BESAA score (*r* = − 0.188, *p <* 0.01). The EAT-26 score was not correlated with the SNS Intensity Scale (*r =* 0.017, *p =* 0.775), but was negatively correlated with the BESAA (*r = −* 0.172, *p =* 0.003). The SNS Intensity Scale score showed no correlation with the BESAA (*r = −* 0.096, *p =* 0.1), but showed a weak positive correlation with the PHQ-9 score (*r =* 0.266, *p <* 0.01). Moreover, scores on the PHQ-9 (*r =* 0.266, *p <* 0.001) and GAD-7 (*r =* 0.242, *p <* 0.001) were positively correlated with the EAT-26 score.

**Table 2:** Correlations among study variables in the Japanese students(*n* = 293)

	1	2	3	4	5	6
BMI	1					
EAT-26	0.042	1				
SNS Intensity Scale	-0.031	0.017	1			
BESAA	-0.188**	-0.172**	-0.096	1		
PHQ-9	-0.001	0.266**	0.157**	-0.295**	1	
GAD-7	-0.01	0.242**	0.082	-0.241**	0.759**	1

Note: BMI: body mass index; EAT-26: Eating Attitudes Scale-26, SNS: social networking services; BESAA: Body-Esteem Scale for Adolescents and Adults; PHQ-9: Patient Health Questionnaire-9; GAD-7: Generalized Anxiety Disorder scale-7

*p < 0.05; **p < 0.01; **p < 0.001

Among the Chinese students, scores on the SNS Intensity Scale (*r =* 0.292, *p <* 0.01), PHQ-9 (*r =* 0.252, *p <* 0.001), and GAD-7 (*r =* 0.217, *p <* 0.001) were positively correlated with the EAT-26 score. Moreover, the BESAA score (*r = −* 0.153, *p =* 0.012) was negatively correlated with the EAT-26 score. Additionally, we investigated the correlations between SNS intensity and body esteem; the BESAA score (*r =* 0.17, *p =* 0.005) was positively correlated with the SNS Intensity Scale score.

### Mediation analyses between Japanese and Chinese students

**Fig. 2:**
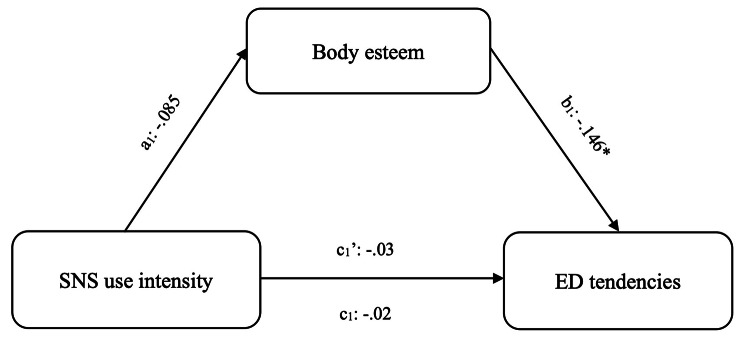
Mediation analyses among Japanese students Note: SNS: social networking services; ED: eating disorders *p < 0.05

**Fig. 3:**
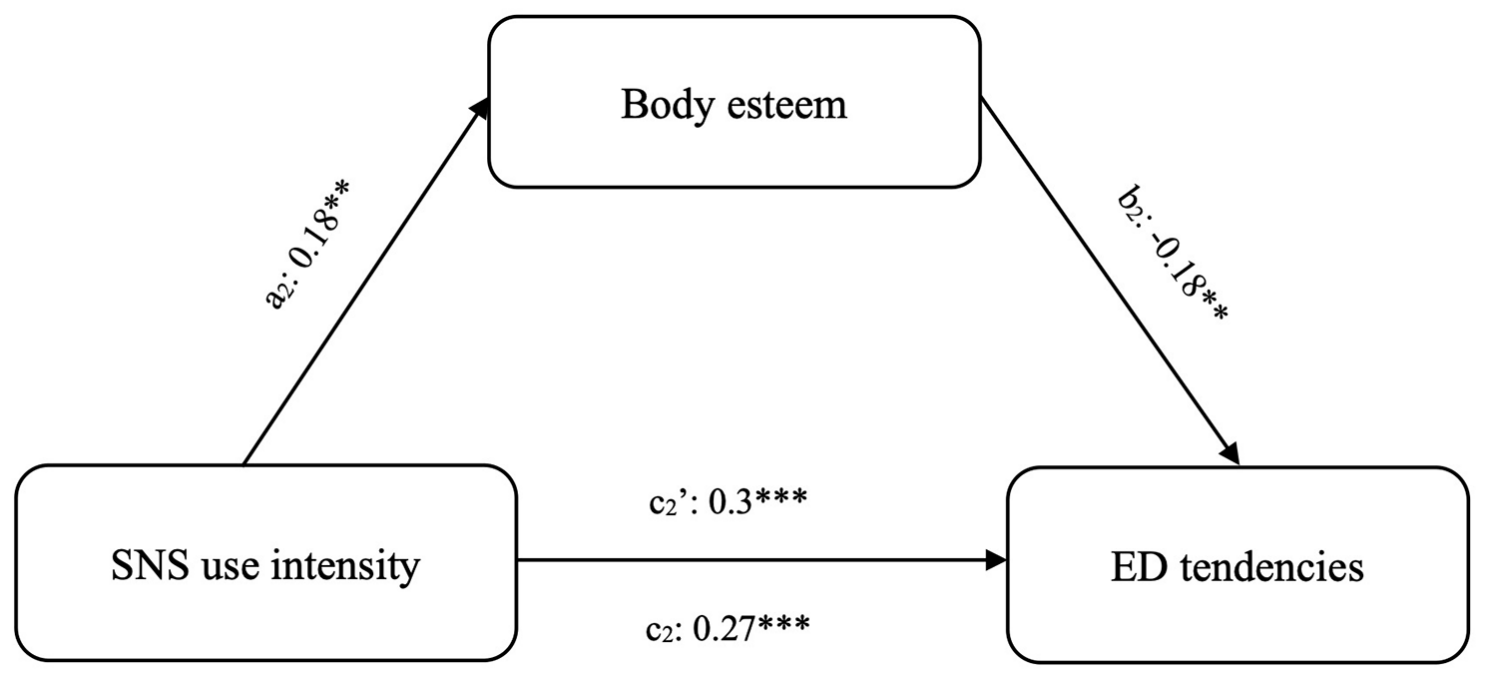
Mediation analyses among Chinese students Note: SNS: social networking services; ED: eating disorders *p < 0.05; **p < 0.01; **p < 0.001

We examined body esteem as a mediator between SNS use intensity and ED tendencies in Japanese (Fig. 2) and Chinese (Fig. 3) students. BMI and gender were included as covariates.

In Japanese students, the indirect effect of SNS use intensity on ED tendencies through body esteem was not significant (*effect* = 0.029, *SE* = 0.022, [*BCa 95% CI* -0.006 to 0.077]). While the effect of SNS intensity on body esteem was not significant (*B =* -0.85), body satisfaction was associated with lower ED tendencies (*B =* -0.146).

In Chinese sample, results from the mediation analyses reveal that indirect effect of.

In Chinese students, the indirect effect of SNS use intensity on ED tendencies through body esteem was significant (*effect =* -0.08, *SE =* 0.04, [BCa 95% CI -0.16 to -0.02]); that is, greater SNS use intensity was associated with greater body esteem (*B =* 0.19), which was associated with lower ED tendencies (*B =* -0.18).

## Discussion

We examined the relationships between ED tendencies, SNS use intensity, and body esteem in Japanese and Chinese students. A total of 564 Japanese and Chinese 18-22-years-olds answered questions on basic demographics and completed the EAT-26, SNS Intensity Scale, BESAA, PHQ-9, and GAD-7.

First, this study (data collected in December 2021) shows that the number of people with a score ≥ 20 was higher (14.7% in Japan and 17% in China) than that in previous studies. A previous study in investigating ED tendencies reported that 8.7% of 494 junior college students in Japan had an EAT-26 score ≥ 20 in 2010 [24]. Another study using the EAT-26 reported a score of ≥ 20 in 59 (4.44%) of 1328 college students in Wuhu, China in 2015 [25]. Since the end of 2019, the coronavirus disease 2019 (COVID-19) pandemic has disrupted people’s daily lives and increased their anxiety, stress, and other negative emotions [26]. Moreover, those with mental disorders, including ED, could be affected in various ways. Therefore, we think the impact of COVID-19 may have resulted in a higher percentage of participants with EAT-26 scores of ≥ 20.

Second, we found gender differences in EAT-26 scores among the Chinese group, consistent with the literature which reporting that women have a higher ED risk than men in Western countries [27, 28]. This possibly reflects that Chinese women are more concerned about their body and weight than Chinese men and Japanese students.

In addition, Japanese women scored higher than Japanese men on the EAT-26, but this difference was not significant. Edman et al. similarly reported that there were no gender differences in the EAT-26 scores among Malaysian university students [29]. Research from Fiji shows that men also experience sociocultural pressure to achieve an ideal muscular body [30]. Thus, our findings suggest that Japanese men are at an equally high risk of developing ED as Japanese women.

The participants spent 31 min to two hours per day on SNS, compared to the average time of 16 min per day on Facebook reported in Japan in 2012 [31], and 15 min to 60 min per day on Weibo reported in China in 2012 [32]. Since 2019, because of the COVID-19 pandemic, many countries have restricted people’s daily activities and movements; for example, work and studying have been shifted to the home as much as possible, which could be one of the reasons for the increased time spent on SNS. Chinese students (*M =* 3.99, *SD =* 1.14) had significantly higher scores than Japanese students (*M =* 3.5, *SD =* 1.71) regarding time spent on SNS. We know that China ended the Zero-COVID policy in December 2022, much later than other countries, resulting in Chinese students spending more time working or studying at home; this may have increased their time spent on SNS. Increased time spent on SNS and the inability to control the use of SNS negatively impact people’s lives [33, 34].

It is worth mentioning that Chinese students reported higher scores on the BESAA than Japanese students. Heine et al. found that self-esteem is lower in Japan than in Western countries [35], but Chinese college students have been known to report high self-esteem, as in Western countries [36, 37]. In China, the only-child policy is currently in effect, and Chinese children receive a great deal of parental attention and care [38], leading to high levels of self-esteem. Low self-esteem has been found to be reciprocally predictive of body dissatisfaction [39, 40]. The results of this study are consistent with our findings that Chinese students generally had higher body esteem than Japanese students.

According to Tables 2 and 3, when we explored the relationship between ED tendencies, SNS use intensity, and body esteem, the correlation coefficients were not high despite a strong correlation (*p <* 0.01). In past studies [41, 42], data with very low correlation coefficients but high correlations such as these have been observer; thus, we used these data.

**Table 3:** Correlations among study variables in the Chinese students(*n* = 271)

	1	2	3	4	5	6
BMI	1					
EAT-26	0.082	1				
SNS Intensity Scale	0.083	0.292^**^	1			
BESAA	-0.026	-0.153*	0.17**	1		
PHQ-9	0.013	0.252**	-0.147*	-0.315**	1	
GAD-7	-0.017	0.217**	-0.025	-0.263**	0.739**	1

Note: BMI: body mass index; EAT-26: Eating Attitudes Scale-26, SNS: social networking services; BESAA: Body-Esteem Scale for Adolescents and Adults; PHQ-9: Patient Health Questionnaire-9; GAD-7: Generalized Anxiety Disorder scale-7

*p < 0.05; **p < 0.01; **p < 0.001

We hypothesized that body esteem mediates the relationship between SNS use intensity and ED tendencies. Unexpectedly, we did not observe this relationship in Japanese students. Although much of the research suggests that frequent SNS use is association with body dissatisfaction, the manner of SNS engagement can also lead to different results. For example, users can search for any content they are interested in; some use SNS to view others’ posts and comment on them or for uploading personal pictures [43]. The way of engagement on SNS has been shown to play an important role in well-being [44]; for example, users who posted statuses with a negative feedback-seeking style were more likely to report body dissatisfaction and ED symptoms [45]. As such, future studies should focus on exploring the relationship between the manner of SNS engagement and ED.

Our findings also showed that greater SNS intensity was associated with higher body esteem, which was associated with lower ED tendencies in Chinese students. This result—a positive relationship between SNS intensity and body esteem in Chinese students—is contrary to most of the literature regarding SNS use and body dissatisfaction. In our study, the SNS Intensity Scale was designed without restrictions on which social media could be used. As we were aware that WeChat’s utilization rate is over 80% in China, we guessed that most Chinese participants would answer the SNS Intensity Scale with reference to WeChat.

WeChat is similar to LINE in Japan, mostly used to communicate with friends, upload photos, and comment on photos. It could be that those who use SNS regularly or who have more friends on SNS are more willing to socialize on social media, get information, or share their own opinions, thereby increasing their self-confidence, including body esteem. SNS is mostly used to post photos or comments; it is also possible that people gained more attention on SNS because of beautiful photos, which helps enhance their self-esteem [46, 47]. We consider this one of the reasons for the positive relationship between SNS intensity and body esteem in the Chinese students.

We did not examine the way individuals engage on SNS; this, together with the lack of clarity regarding which SNS was used, was among the main limitations of this study. In addition, findings should be understood in the context to several limitations. We asked Cross Marketing Inc. for distribution and collection, but the subjects recruited through this channel were those who have registered with this company, and it is not representative of the entire college student population. In the population, this study used convenience sample, and recruited subjects nationwide, but the sample size was relatively small and age range was narrow. Future research on this topic should incorporate a large, more diverse participant sample. In the methodology, this study was self-report questionnaire, which is a convenient method but carries the risk that the subjects did not make authentic completions.

Additionally, at the time of data collection, 29 individuals had a BMI > 30 kg/m^2^, and some Chinese students weighed up to 190 kg. In China, an alternative unit of measurement of weight is “catty” (1 kg = 2 catty). Therefore, we speculate that these unusual weight data are likely owing to the fact that the participants did not see that the weight was marked in kg when filling out the questionnaire, and mistakenly filled in the weight according to the “catty” unit of measurement.

The relationship between SNS use, ED, and body dissatisfaction was compared between the two Asian countries of Japan and China. This study also included men, adding to the scant evidence pertaining to men. First, the results for the Japanese and Chinese students indicate that the status of ED in Asian countries is gradually converging with that in Western countries. Most existing research on ED has focused on Western countries. Chinese students, whose access to Western media and influences is lower than that of Japanese students, reported more ED tendencies and a stronger association between SNS use and ED tendencies. Therefore, it is important to provide evidence of the relationship between SNS use and ED in Asian countries, especially those such as China with their unique SNS platforms.

Second, the ratio of studies on ED related to men and women has been 1:10; therefore, there is little data on men. However, owing to the aesthetic culture espoused on SNS, men are also becoming conscious of their body shape and weight, which increases their risk of developing ED. Therefore, it is extremely important to consider men in ED studies.

## Conclusion

This study provides evidence for an international comparison between Asian countries on the relationship between ED and SNS use. According to our findings, Japanese and Chinese students experience similar levels of the ED tendencies.

Japanese and Chinese students reported the opposite correlation between SNS use intensity and body esteem. The results suggest that more SNS use does not necessarily lead to body dissatisfaction. However, Tiggemann et al. reported that appearance-based SNS use was associated with body dissatisfaction and ED [8]. This is one of the limitations of this study and implies that in future studies that examine the relationship between SNS use and body esteem, it is necessary to include factors related to appearance comparisons.

In addition, we noticed that men in Japan reported similar levels of EAT-26 scores as women. We suggest conducting more studies that investigate gender differences in ED tendencies in Japan in future.

## Data Availability

The datasets used and analyzed during the current study are available from the corresponding author upon reasonable request.

## Abbreviations

AN: Anorexia nervosa
BED: Binge eating disorder
BESAA: Body Esteem Scale for Adolescents and Adults
BMI: Body mass index
BN: Bulimia nervosa
COVID-19: Coronavirus disease 2019
EAT-26: Eating Attitudes Test-26
ED: Eating disorders
GAD-7: Generalized Anxiety Disorder-7
PHQ-9: Patient Health Questionnaire-9
SNS: Social Networking Services
